# Analytical solutions of the radiative transport equation for turbid and fluorescent layered media

**DOI:** 10.1038/s41598-017-02979-4

**Published:** 2017-06-19

**Authors:** André Liemert, Dominik Reitzle, Alwin Kienle

**Affiliations:** Institut für Lasertechnologien in der Medizin und Meßtechnik an der Universität Ulm, Helmholtzstr., 12, D-89081 Ulm, Germany

## Abstract

Accurate and efficient solutions of the three dimensional radiative transport equation were derived in all domains for the case of layered scattering media. Index mismatched boundary conditions based on Fresnel’s equations were implemented. Arbitrary rotationally symmetric phase functions can be applied to characterize the scattering in the turbid media. Solutions were derived for an obliquely incident beam having arbitrary spatial profiles. The derived solutions were successfully validated with Monte Carlo simulations and partly compared with analytical solutions of the diffusion equation.

## Introduction

The radiative transport equation (RTE) is the standard equation for describing particle propagation in many different research areas such as neutron transport in reactor physics^1^ or light transport, e.g., in astronomy, in atmospheric physics, and in biophotonics^2, 3^. Commonly, the RTE is solved using numerical methods, e.g., with the finite volume method^4^ or with Monte Carlo simulations^5^, due to the lack of analytical solutions. Recently, however relevant analytical solutions were obtained for the infinite scattering medium by applying the *P*_*N*_ method and the method of rotated reference frames^6, 7^. Solutions for semi-infinite (or slab) media were reported for refractive index matched boundary conditions^8, 9^. Recently, we solved the radiative transport equation for the case of semi-infinite media and mismatched boundary conditions^10^. We furthermore verified the analytical solutions of the radiative transport equation by comparison with Monte Carlo simulations^11^. This model was, in addition, applied to the spatial frequency domain^12^. Also in this domain the derived analytical solutions were successfully verified against numerical solutions.

Solutions of the RTE for homogeneous media are often used to describe the light propagation in scattering media. An important application is the solution of the inverse problem, the determination of the optical properties of the investigated medium. However, in many cases it is an invalid approximation to describe the considered scattering medium as macroscopically homogeneous. Instead, for many scattering media the assumption of a layered geometry is much more precise. Examples in the field of biophotonics are the layers of the skin (e.g., epidermis and dermis), the layers of the limbs (e.g. skin, subcutaneous fat, and muscle) or the layers of the head (e.g. skin, subcutaneous fat, scull, cerebrospinal fluid, and brain matter). Due to the fact that retrieving the optical properties of layered media using numerical methods is inefficient (e.g. with non-linear regression^13^ or with look-up-tables^14^) and due to the lack of analytical solutions of the RTE for these geometries, analytical solutions of the diffusion equation^15^, an approximations to the RTE, are often applied. However, the diffusion theory has several shortcomings not only at short time scales and large spatial frequencies, but also far away from the incident source term, if layered scattering media are considered, see Results section.

Similar arguments as presented in the previous paragraph are true for describing the light propagation of fluorescence light which is important for a lot of applications, e.g., in microscopy or drug investigations in small animal studies^16, 17^. For the case of fluorescing scattering media no analytical solution of the RTE was reported neither for homogeneous nor for layered media.

In this study we present the solutions of the RTE for multi-layered turbid media. These solutions comprise arbitrary incident beam profiles having arbitrary incident angles and, similarly, arbitrary solid angles for detection. Further, arbitrary rotationally symmetric scattering functions can be treated such as the Henyey-Greenstein function^18^, the Reynolds-McCormick function^19^, or scattering functions obtained by Mie-theory^20^. Arbitrary refractive indices can be chosen for each layer which are calculated by the exact Fresnel formulae. Solutions are obtained for all spatial frequency domains (spatial frequency steady state domain, spatial frequency temporal frequency domain, and spatial frequency time domain) and in all spatial domains (steady-state domain, temporal frequency domain, and time domain). Moreover, solutions for all relevant quantities like the fluence, the radiance, the reflected and transmitted light are derived. Furthermore, the bottom layer can be chosen to be finite or infinitely thick. In addition, all above mentioned solutions are not only derived for elastically scattered light but also for fluorescence. The derived equations are compared to Monte Carlo simulations for scattering media with up to three layers showing an excellent agreement within the inherent statistical noise of the numerical method.

## Theory

### Analytical solutions of RTE

The radiative transport equation

1 $$\hat{{\bf{s}}}\cdot \nabla I({\bf{r}},\hat{{\bf{s}}})+{\mu }_{t}I({\bf{r}},\hat{{\bf{s}}})={\mu }_{s}\int I({\bf{r}},\hat{{\bf{s}}}^{\prime} )f(\hat{{\bf{s}}}\cdot \hat{{\bf{s}}}^{\prime} )\,{{\rm{d}}}^{2}s^{\prime} +S({\boldsymbol{\rho }})\delta (z)\delta (\hat{{\bf{s}}}-\hat{{\bf{z}}})$$

is usually applied to predict the light propagation in mesoscopic and macroscopic scattering media^3, 21^. In the following, *μ*_*t*_ = *μ*_*a*_ + *μ*_*s*_ is the total attenuation coefficient, *μ*_*a*_ is the absorption coefficient, *μ*_*s*_ denotes the scattering coefficient and *S*(***ρ***) is the lateral beam profile. In this report we show results for a Gaussian beam corresponding with $S(\rho )=2/(\pi {\rho }_{w}^{2})\,\exp (-2{\rho }^{2}/{\rho }_{w}^{2})$, where *ρ*_*w*_ denotes the radius of the beam. The unit vector $\hat{{\bf{s}}}=(\mu ,\varphi )$ specifies the direction of the photon propagation and $f(\hat{{\bf{s}}}\cdot \hat{{\bf{s}}}^{\prime} )$ is the scattering phase function.

The analytical approach for solving the three-dimensional RTE is based on the Fourier transform regarding the lateral coordinates as well as on the modified spherical harmonics method regarding the angular coordinates^9^. The resulting eigenvalue problem is solved via an eigenvalue decomposition of a symmetric tridiagonal matrix, which has to be performed once only. The associated angular dependent boundary conditions are implemented in form of the Marshak-type conditions. In this chapter, we briefly sketch the applied solution approach for the case of a two-layered medium, whereas numerical results are shown and verified for up to three layers. A detailed derivation of the complete analytical solution containing all calculation steps will be presented in a future work. The outlined approach can also be extended for modeling the photon transport within media consisting of an arbitrary number of different layers in a straight forward manner. Here it is assumed that the last layer extends to infinity, but again a generalization to a slab geometry can easily be performed. Results for both the reflectance and the fluorescence are given for mismatched boundary conditions. For the special case of calculations in order *P*_3_ the solutions were obtained in explicit form similar to those described earlier for a semi-infinite medium^22^. The exact boundary conditions using Fresnel’s formulae were used both for the boundary to the nonscattering medium as well as for the boundaries between the different scattering layers. In detail, the radiance at the boundary *z* = 0 must satisfy the boundary condition

2 $${I}_{1}({\boldsymbol{\rho }},z=0,\mu ,\varphi )=R(\mu ){I}_{1}({\boldsymbol{\rho }},z=0,-\mu ,\varphi ),$$

where *μ* > 0 and *R*(*μ*) is the probability for reflection at the surface calculated with Fresnel’s formulae. Moreover, the radiance at an interface at *z* = *L* between two layers must satisfy the following two conditions

3 $$\begin{array}{rcl}{I}_{1}({\boldsymbol{\rho }},L,-\mu ,\varphi ) & = & {R}_{1}(\mu ){I}_{1}({\boldsymbol{\rho }},L,\mu ,\varphi )\\  &  & +\frac{{n}_{1}^{2}}{{n}_{2}^{2}}(1-{R}_{1}(\mu )){I}_{2}({\boldsymbol{\rho }},L,-\sqrt{1-\frac{{n}_{1}^{2}}{{n}_{2}^{2}}(1-{\mu }^{2})},\varphi ),\end{array}$$

4 $$\begin{array}{rcl}{I}_{2}({\boldsymbol{\rho }},L,\mu ,\varphi ) & = & {R}_{2}(\mu ){I}_{1}({\boldsymbol{\rho }},L,-\mu ,\varphi )\\  &  & +\frac{{n}_{2}^{2}}{{n}_{1}^{2}}\mathrm{(1}-{R}_{2}(\mu )){I}_{1}({\boldsymbol{\rho }},L,\sqrt{1-\frac{{n}_{2}^{2}}{{n}_{1}^{2}}\mathrm{(1}-{\mu }^{2})},\varphi ),\end{array}$$

defined for *μ* > 0 with

5 $$\begin{array}{rcl}{R}_{1}(\mu ) & = & \frac{1}{2}{(\frac{{n}_{2}\mu -{n}_{1}\sqrt{1-{({n}_{1}/{n}_{2})}^{2}\mathrm{(1}-{\mu }^{2})}}{{n}_{2}\mu +{n}_{1}\sqrt{1-{({n}_{1}/{n}_{2})}^{2}\mathrm{(1}-{\mu }^{2})}})}^{2}\\  &  & +\frac{1}{2}{(\frac{{n}_{2}\sqrt{1-{({n}_{1}/{n}_{2})}^{2}\mathrm{(1}-{\mu }^{2})}-{n}_{1}\mu }{{n}_{2}\sqrt{1-{({n}_{1}/{n}_{2})}^{2}\mathrm{(1}-{\mu }^{2})}+{n}_{1}\mu })}^{2}.\end{array}$$

In this context, the indices 1 and 2 refer respectively to the upper und the lower layers at an interface inside the layered medium. The function *R*_2_(*μ*) can be directly obtained from *R*_1_(*μ*) via interchanging the indices 1 and 2. Arbitrary rotationally symmetric scattering functions can be handled, which may be different for each layer. Solutions for obliquely (and perpendicularly) incident beams with different spatial profiles were found for the reflectance $R(\rho )=-\int \mu I({\boldsymbol{\rho }},0,\hat{{\bf{s}}})\,{{\rm{d}}}^{2}s$, the internal fluence ${\rm{\Phi }}({\bf{r}})=\int I({\bf{r}},\hat{{\bf{s}}})\,{{\rm{d}}}^{2}s$ as well as for the radiance. In the case of the fluorescence, one has to solve the following coupled equations

6 $$\begin{array}{rcl}\hat{{\bf{s}}}\cdot \nabla {I}_{x}({\bf{r}},\hat{{\bf{s}}})+{\mu }_{tx}{I}_{x}({\bf{r}},\hat{{\bf{s}}}) & = & {\mu }_{sx}\int {I}_{x}({\bf{r}},\hat{{\bf{s}}}^{\prime} )f(\hat{{\bf{s}}}\cdot \hat{{\bf{s}}}^{\prime} )\,{{\rm{d}}}^{2}s^{\prime} +S({\boldsymbol{\rho }})\delta (z)\delta (\hat{{\bf{s}}}-\hat{{\bf{z}}}),\\ \hat{{\bf{s}}}\cdot \nabla {I}_{m}({\bf{r}},\hat{{\bf{s}}})+{\mu }_{tm}{I}_{m}({\bf{r}},\hat{{\bf{s}}}) & = & {\mu }_{sm}\int {I}_{m}({\bf{r}},\hat{{\bf{s}}}^{\prime} )f(\hat{{\bf{s}}}\cdot \hat{{\bf{s}}}^{\prime} )\,{{\rm{d}}}^{2}s^{\prime} +\frac{{\mu }_{ax}}{4\pi }{{\rm{\Phi }}}_{x}({\bf{r}}),\end{array}$$

where the subscripts *x* and *m* refer to the excitation and emission wavelengths of the light. The solutions of these equations are considerably involved. However, we note that, if the reader is interested in the code, the corresponding author can be contacted. The solutions of the RTE, as well as those of the diffusion equation used in this work^15^, are based on a 2-D Fourier transform in space and a Laplace transform in time. In order to obtain solutions in the spatial domain or in the time domain, these transforms must be numerically inverted. If, as for the results presented, rotationally symmetric problems are considered, the 2-D Fourier transform can be replaced by the one-dimensional Hankel transform.

For the solutions of the diffusion equation^23^, we used a Laplace inversion method based on a line integration along a hyperbolic contour that was published recently^24^. Unfortunately, this method is not applicable to the RTE solutions. We therefore used a different line-integration based method with a two-part contour that can avoid the integration over branch-cuts for the RTE solution while retaining the advantage of time-independent evaluation points. This method will be published in a future work.

### Monte Carlo simulations

In order to validate the derived analytical solutions for different orders of the *P*_*N*_ method we compared them with results obtained with the Monte Carlo method. This numerical method is most often applied to solve the RTE for applications in the field of biophotonics. In our code mainly standard procedures were implemented to calculate the light propagation in the scattering medium. For example, Fresnel’s equations were solved at the boundary between the scattering medium and the non-scattering surrounding. No weighting schemes were applied. The calculation of the fluorescence light propagation was implemented directly as described earlier^25^. Thus, convolution algorithms were avoided. The code was successfully validated against other Monte Carlo codes written in our group.

## Results

In this chapter we show results of the reflectance in the spatial steady-state and in the spatial time domain of the derived analytical solutions and compare them to Monte Carlo simulations and partly to the diffusion theory. Especially, we show the accuracy of the solutions for different orders *N* of the *P*_*N*_ method. The spatially resolved steady-state reflectance is denoted by *R*_*s*_, whereas *R*_*t*_ denotes the time-resolved reflectance. These two quantities are connected by

7 $${R}_{s}(r)={\int }_{0}^{\infty }\,{R}_{t}(r,t)\,{\rm{d}}t=\tilde{R}({\rm{r}},{\rm{s}}=0).$$

**Figure 1:**
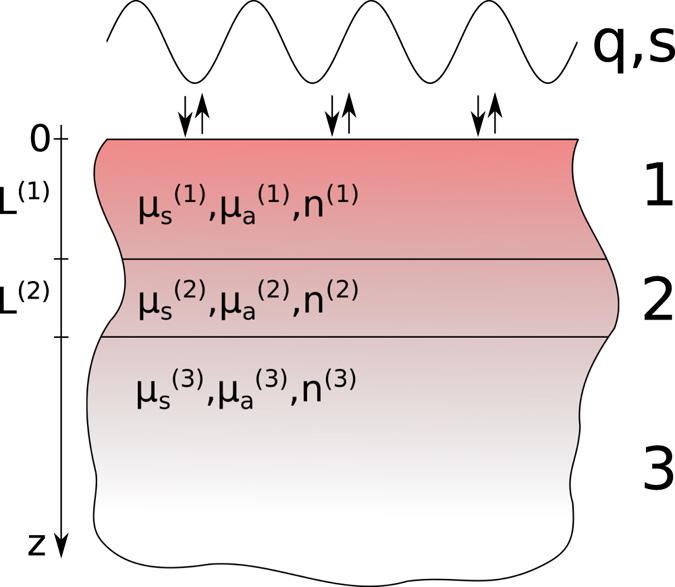
Schematic of the considered three-layered medium. The solution of the RTE is calculated in the Laplace and spatial frequency domains. By numerically inverting these transforms, solutions in the spatial domain and time domain can be obtained. For the fluorescence solution, an additional set of optical parameters for the fluorescence wavelength is assigned to each layer.

We considered two different generic multi-layered models for human tissue. The first one is a two-layered model. For the upper layer with varying thickness, which e.g. represents skin including the subcutaneous fat layer, optical properties of ${\mu }_{a}^{\mathrm{(1)}}=0.02\,{{\rm{mm}}}^{-1}$ and ${\mu }_{s}^{^{\prime} \mathrm{(1)}}=2.0\,{{\rm{mm}}}^{-1}$ are used. For the lower semi-infinite layer (e.g. muscle tissue), we used ${\mu }_{a}^{\mathrm{(2)}}=0.03\,{{\rm{mm}}}^{-1}$ and ${\mu }_{s}^{^{\prime} \mathrm{(2)}}=0.5\,{{\rm{mm}}}^{-1}$. The second, three-layered model consists of an upper layer (e.g. skin) with ${\mu }_{a}^{\mathrm{(1)}}=0.02\,{{\rm{mm}}}^{-1}$, ${\mu }_{s}^{^{\prime} \mathrm{(1)}}=2.0\,{{\rm{mm}}}^{-1}$ and thickness *L* ^(1)^ = 1 mm, a middle layer representing e.g. subcutaneous fat tissue with ${\mu }_{a}^{\mathrm{(2)}}=0.003\,{{\rm{mm}}}^{-1}$, ${\mu }_{s}^{^{\prime} \mathrm{(2)}}=1.0\,{{\rm{mm}}}^{-1}$ and thickness *L* ^(2)^ = 2 mm and again a lower semi-infinite layer with ${\mu }_{a}^{\mathrm{(3)}}=0.02\,{{\rm{mm}}}^{-1}$ and ${\mu }_{s}^{^{\prime} \mathrm{(3)}}=0.5\,{{\rm{mm}}}^{-1}$ representing e.g. muscle tissue. For all layers, a refractive index of *n*_*i*_ = 1.4 is assumed and the surrounding medium is set to air with *n*_*e*_ = 1.0. A schematic of this model is shown in Fig. 1. If not stated otherwise, all calculations use the Henyey-Greenstein phase function with an anisotropy factor of *g* = 0.8 and a Gaussian beam profile with a beam radius of *ρ*_*w*_ = 0.5 mm. For time-resolved calculations, an infinitely short pulse is used, spatially resolved calculations are steady-state solutions.

**Figure 2:**
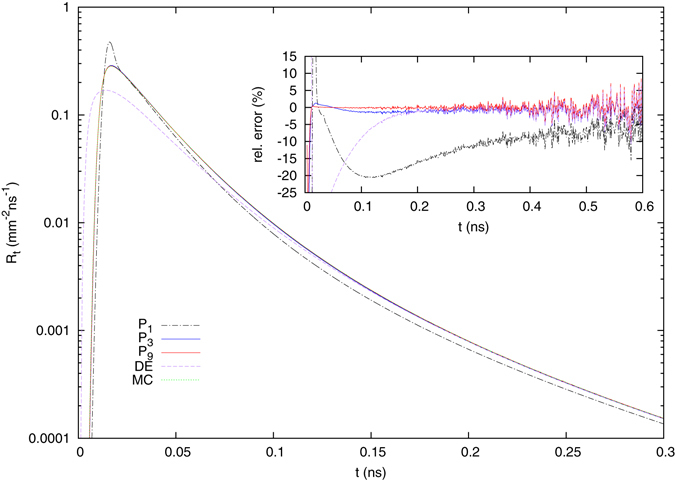
Time-resolved reflectance from the two-layered scattering medium with upper layer thickness of *l* = 2 mm and a refractive index of *n*_*m*_ = 1.4 due to an infinitely short, perpendicularly incident light pulse with a spatial Gaussian beam profile. The beam radius is *ρ*_*w*_ = 0.5 mm and the source-detector separation *ρ* = 2 mm. The refractive index of the surrounding medium is *n*_0_ = 1.0. Already for *N* ≥ 3, a good agreement between the Monte Carlo simulation and the *P*_*N*_ solution is observed. For the Monte Carlo simulation 10^11^ photons were calculated.

We begin with the time resolved reflectance from the two-layered model. Figure 2 shows the results for the *P*_1_, *P*_3_ and *P*_9_ approximations together with the corresponding solution of the diffusion equation and a Monte Carlo simulation. The inset shows the relative error of the *P*_*N*_ approximations, which is calculated as $({R}_{x}-{R}_{MC})/{R}_{MC}$. For *N* ≥ 3, the *P*_*N*_ solutions agree well with the Monte Carlo simulation having errors of about 1% or less. As expected, the diffusion theory also predicts the reflectance well for large times $t\mathop{ > }\limits_{ \tilde {}}150\,{\rm{ps}}$, but fails for smaller times due to the diffusion approximation, which cannot describe the influence of the phase function and does not fulfill the finite speed of light. The *P*_1_ approximation is clearly inferior to the much simpler diffusion theory and should not be used in this case.

Based on this and further comparisons, we conclude that the *P*_3_ approximation is sufficient for most time-resolved reflectance calculations with multi-layered models, as was found previously for semi-infinite systems^22^. Especially when using small source-detector separations to extract more information about the upper layer or to achieve a better lateral resolution in diffuse optical tomography^26^, the *P*_3_ approximation and higher order approximations are vastly superior to the diffusion theory.

**Figure 3:**
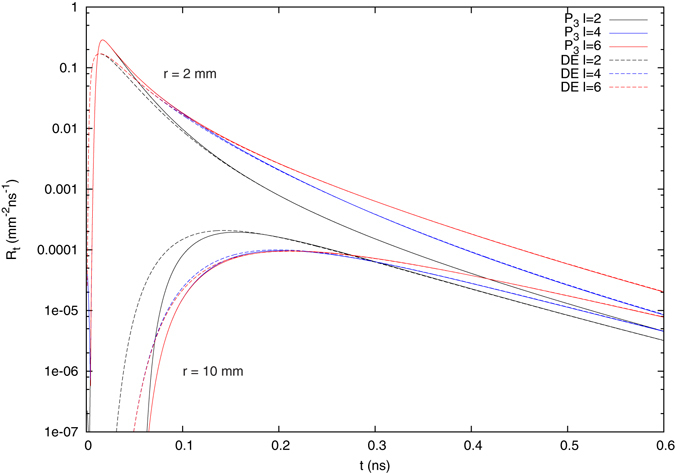
Comparison of time-resolved reflectance results produced by the *P*_3_ approximation and diffusion theory for different upper layer thicknesses of the two-layered model with two source-detector distances *r*.

Figure 3 shows a comparison between the *P*_3_ approximation and the diffusion theory for different thicknesses of the upper layer and two different source-detector separations *r*. For the large measurement distance of *r* = 10 mm, the diffusion approximation is valid once the reflectance drops after its initial peak. However, for this distance, the curves change significantly for all times, when the upper layer thickness is changed. When solving the inverse problem, this can lead to difficulties in separating the optical properties of the layers, if the upper layer is thin compared to the source-detector separation. In contrast, for the small measurement distance *r* = 2 mm, all curves coincide for short times, meaning that the reflectance in this range is mainly governed by the optical properties of the upper layer. This makes the determination of the individual optical parameters much easier, particularly if used in conjunction with an additional large separation measurement. For such short times however, the diffusion approximation fails and produces incorrect results for a large part of the three orders of magnitude from the initial peak, that are typically measured in time resolved experiments. As shown in Fig. 2, the *P*_*N*_ approximations, even for order *N* = 3, offer a substantial improvement for this kind of problems.

**Figure 4:**
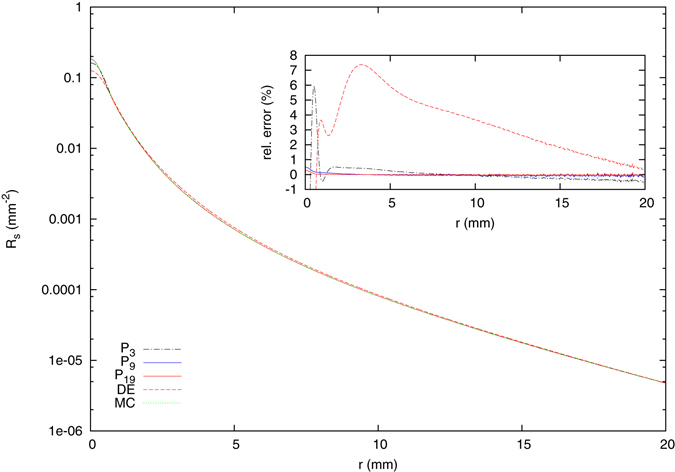
Steady-state spatially resolved reflectance from the three-layered scattering medium with an absorption coefficient of the lower semi-infinite layer of ${\mu }_{a}^{\mathrm{(3)}}=0.02\,{{\rm{mm}}}^{-1}$ and a refractive index of *n*_*m*_ = 1.4 due to a perpendicularly incident Gaussian beam with a beam radius of *ρ*_*w*_ = 0.5 mm. The refractive index of the surrounding medium is *n*_0_ = 1.0. For *N* ≥ 9, the Monte Carlo simulation and the *P*_*N*_ solution agree well. For the Monte Carlo simulation, 10^11^ Photons were calculated.

**Figure 5:**
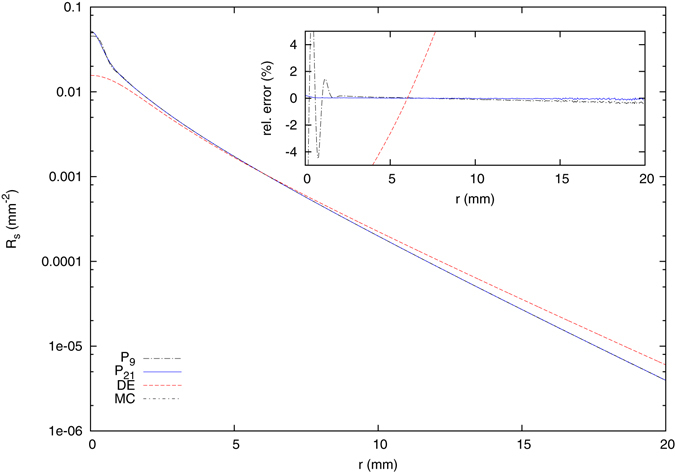
Steady-state spatially resolved reflectance from the three-layered scattering medium, but with interchanged scattering coefficients ${\mu }_{s}^{^{\prime} \mathrm{(1)}}=0.5\,{{\rm{mm}}}^{-1}$ and ${\mu }_{s}^{^{\prime} \mathrm{(3)}}=2.0\,{{\rm{mm}}}^{-1}$. All other parameters are equal to those of Fig. 4. Due to the large transport length in the thin upper layer, the DE solution becomes unusable, whereas the multi-layer RTE solution still produces accurate results.

Next, we considered the steady-state, spatially resolved reflectance from the three-layered skin model. Figure 4 shows the results of the *P*_3_, *P*_9_ and *P*_19_ solutions again together with the diffusion approximation, a Monte Carlo simulation and an inset containing the relative errors regarding the simulation. For *N* ≥ 9, the *P*_*N*_ solutions agree well with the Monte Carlo simulation, whereas the solution of the diffusion equation shows larger differences. We note, first, that these differences of the diffusion equation are relatively small due to the use of the Henyey-Greenstein phase function with a relatively large anisotropy factor. The use of other phase functions results usually in much larger errors, see below. However, even for large distances, well within the so called diffusion regime, the solution of the diffusion equation is not approaching the solution of the radiative transport theory. Instead, the solutions intersect (at about 22 mm) and the relative error grows with increasing distance (not shown). We investigated this behavior more thoroughly and found that it depends e.g. on reduced scattering coefficient of the considered medium. If, as an example, the scattering coefficients of the first and third layer of the system considered here are interchanged, the diffusion approximation breaks down entirely due to the larger mean free path in the (thin) upper layer. This is shown in Fig. 5.

If a high accuracy is required, this behaviour of the diffusion equation can be a problem even, if the transport length is not, as it is the case here, larger than the layer thickness. Contrarily, the RTE solution performs much better. Unsurprisingly though, the approximation order that is required for a given accuracy rises, if the radiance inside the medium becomes more peaked. In extreme cases, the numerical evaluation of the RTE solution can then become quite challenging.

**Figure 6:**
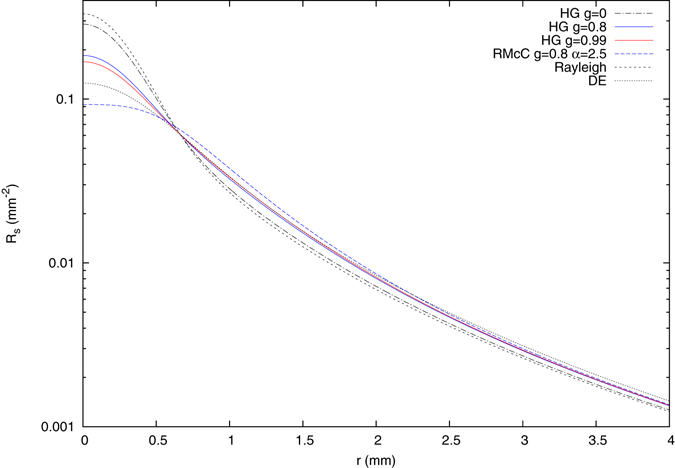
Steady-state spatially resolved reflectance from the three-layered scattering medium using the *P*_9_ approximation with different phase functions.

Next, we show the reflectance for the initial three-layered model with different phase functions. These include the Henyey-Greenstein (HG) phase function with anisotropy factors of *g* = 0, *g* = 0.8 and *g* = 0.99, the Reynolds-McCormick (RMcC) phase function with *g* = 0.8 and *α* = 2.5 and the Rayleigh phase function. Figure 6 shows the *P*_9_ results for short distances, where the effect of the phase function is most prominent, together with the diffusion theory result for comparison. Although the ${\mu }_{s}^{^{\prime} }$-values for all curves are the same, the reflectance varies significantly and the diffusion equation does not approximate the reflectance for any of the considered phase functions well. This again shows that the *P*_*N*_ solution improves strongly the determination of optical parameters of individual layers in multi-layered systems.

**Figure 7:**
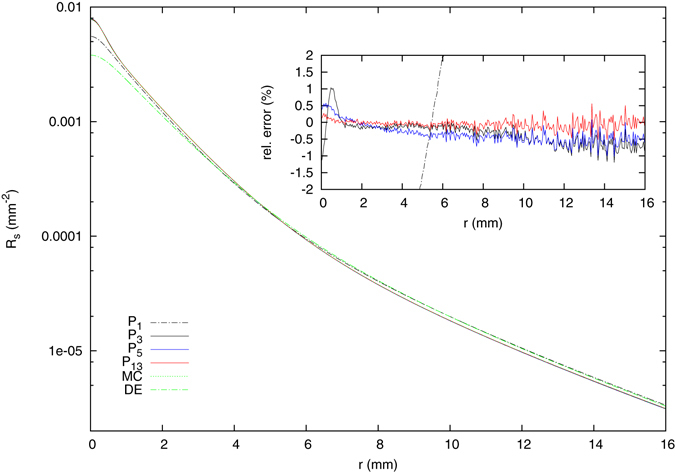
Steady-state spatially resolved fluorescence from the two-layered scattering medium with an upper layer thickness of *l* = 2 mm. Only the upper layer is fluorescent with a quantum yield of ${{\rm{\Phi }}}_{e}^{\mathrm{(1)}}=1.0$. The system is illuminated by a Gaussian beam with radius *ρ*_*w*_ = 0.5 mm. The optical properties of the layers were assigned to the excitation wavelength, whereas those of the fluorescence wavelength were chosen to be 20% below their respective excitation counterparts. The phase function and the boundary conditions are identical for both wavelengths. The *P*_3_ solution already shows a very good agreement with the Monte Carlo simulation. For the Monte Carlo simulation, 8 · 10^9^ Photons were calculated.

Finally, we considered the spatially resolved fluorescence of the two-layered system. Here, the optical parameters of all layers can be chosen differently for the excitation and the fluorescence wavelengths. We assigned the parameters of the model mentioned above for a two-layered medium to the excitation wavelength and chose those for the fluorescence wavelength to be 20% lower than their respective counterparts for the excitation. The phase function and the boundary condition were the same for both wavelengths and the refractive index of the medium is assumed to be *n*_*m*_ = 1.4, independent of the wavelength. The fluorescence quantum yield ${{\rm{\Phi }}}_{e}$ can be set individually for each layer. We set ${{\rm{\Phi }}}_{e}^{\mathrm{(1)}}=1.0$ and ${{\rm{\Phi }}}_{e}^{\mathrm{(2)}}=0$, meaning that only the upper layer is fluorescent. Figure 7 shows the fluorescence for the *P*_*N*_ approximation for different orders *N* together with a Monte Carlo simulation and the solution of the diffusion equation. The inset again shows the relative errors with respect to the simulation. Already for *N* ≥ 3, a very good agreement is observed. Since the fluorescence is calculated using the excitation absorption rate inside the whole medium as isotropic sources, the approximation order required to achieve a given accuracy is much lower for the fluorescence than for the reflectance of a collimated incident beam. In this case we also included the results for the *P*_1_ approximation, which turns out to be considerably closer to the correct solution of the RTE than the solution of the diffusion equation, although the underlying equations for *P*_1_ approximation and the diffusion theory are identical except for the imposed boundary conditions and the source term.

We close this section with a few remarks on the calculation time for the presented multi-layered RTE solution. Table 1 shows the calculation times for the spatial domain steady state solutions shown in Figs 4 and 6 with 2001 spatial evaluation points. The Fresnel coefficients are calculated and stored in advance. Therefore, the refractive indices are fixed. We then distinguish two calculation times. The first one called “full calculation” is the time for the calculation of a single curve. The second one, “recalculation”, is the time for the computation of a second curve, where all optical parameters (except for the refractive indices) of the medium are changed, but the evaluation points and the input beam properties are kept constant. This allows caching and reusing some intermediate results from the full calculation. Since this is the typical scenario when solving an inverse problem, where speed matters most, this is the more important number.

**Table 1:** Calculation times for the steady-state spatial domain RTE solution in double precision for 2001 spatial conditions.

	Half-space		3-layered	
	Full calculation	Recalculation	Full calculation	Recalculation
*P*_3_	15.2 ms	0.85 ms	18.7 ms	4.3 ms
*P*_9_	73 ms	58 ms	318 ms	304 ms

The calculations for Table 1 were performed on a standard desktop PC equipped with an Intel^®^ Core™ i7-920 processor in double precision. It has to be noted that double precision is not always sufficient to accurately compute the RTE solution, especially if high approximation orders and low scattering coefficients compared to 1/*ρ*_*w*_ are used. In this work, double precision was always sufficient, except for the calculations in Fig. 5. These multi-precision calculations are usually very expensive. As an example, the calculation time for the three-layered *P*_9_ solution increases to about 5 s using quad precision (implemented as double-double arithmetic) for the present algorithm.

## Conclusions and Discussion

Analytical solutions of the RTE, the fundamental equation for description of the light propagation in random media in the mesoscopic and macroscopic scales, were derived for the first time for layered media. The analytical solutions were not only found for elastically scattered light but also for fluorescence light. In general, fluorescence imaging is an important method in life science^27^, but up to now there was no analytical solution of the RTE available even for the simpler case of a homogeneous medium.

The analytical solutions were derived using the *P*_*N*_-method und were compared to numerical solutions of the RTE applying Monte Carlo simulations. It was shown that for all cases the analytical solutions agree with the numerical solutions provided that the approximation order *N* was sufficiently high. Further, solutions with different orders *N* were compared and the differences to the exact solutions were shown. It was found that for a lot of applications especially in the time domain and for fluorescence a low order approximation is adequate.

The comparisons were performed for typical optical and geometrical parameters encountered in biophotonics such as for non-invasive hemodynamic measurements on the forearm. However, possible applications are manifold in a variety of fields reaching from atmospheric optics to applications in process control, e.g., in the pharmaceutical or food industry. Results were shown in the spatial and in the time domains, but the solutions are obtained in all domains. Furthermore, the derived analytical solutions can be easily applied for other applications. For example, first, the solutions for the reflectance can be extended to solve the correlation transport equation^28^, with which moving particles in scattering media can be characterized using correlation measurements, Laser Doppler experiments, or Laser speckle measurements^29^. A possible application is the measurement of the blood hemodynamics in the human brain, where it is important to consider the layered structure of the head. Second, the solutions for fluorescence light can also be used for other inelastic processes like Raman scattering. Third, the fluence rate in layered media calculated with the analytical solutions are e.g. important for applications in photoacoustic tomography^30^, where diffusion theory delivers usually inaccurate results.

Finally we note that the calculation time of the derived analytical solutions for layered media in the spatial frequency domain at a single spatial frequency is considerably shorter (roughly three orders of magnitude) than the already short processing times in the spatial domain shown in Table 1, because the analytical solutions are first derived in the spatial frequency domain, and are, then, transformed into the spatial domain. This is especially interesting because measurements in the spatial frequency domain are an emergent imaging technology characterizing quantitatively the structure and chemical content of turbid media like biological tissue^31^.
